# PAs in the National Guard and Reserves: Army, Navy, Air Force, Coast Guard

**DOI:** 10.1097/01.JAA.0000742984.47706.b9

**Published:** 2021-06-24

**Authors:** Roderick S. Hooker, Andrzej Kozikowski, Johnny Paul

**Affiliations:** **Roderick S. Hooker** is a health services researcher and an adjunct professor of health policy at Northern Arizona University's Phoenix Biomedical Campus. **Andrzej Kozikowski** is director of research at the National Commission on Certification of Physician Assistants in Johns Creek, Ga. **Col. Johnny Paul** is chair of the Department of Combat Medic Training at Fort Sam Houston in Texas. The authors have disclosed no potential conflicts of interest, financial or otherwise.

**Keywords:** COVID-19, disaster response, primary care, deployment, pandemic, armed forces

## Abstract

The role of physician assistants (PAs) in the United States extends to the Army National Guard; Air National Guard; and reserves of the Army, Navy, Air Force, and Coast Guard (collectively known as reserve components). To understand the duality of civilian-military PA roles, a census of the armed forces was undertaken, drawing on knowledgeable senior PA medical officers in each of the services. The survey was supplemented with data from the National Commission on Certification of Physician Assistants. In 2020, there were 1,944 PAs in the five military reserve components with the majority (1,597) in the Army. Most National Guard, Air National Guard, and Reserve PAs fill medical officer roles, drill with units, and are subject to active duty. As soldiers, sailors, and airmen, military PAs are trained in health, safety, warfare readiness, casualty, trauma, and crisis response. The tenure of a reserve component PA in the military ranged between 10.2 and 17.8 years. In their civilian roles, most PAs are licensed and clinically active—the majority report they work in family/general medicine, emergency medicine, general surgery, or orthopedic medicine and surgery. This dual-career role and responsibility suggests the utility and flexibility of the PA is broader than previously reported. The findings set the stage for additional research on healthcare professionals during times of domestic and international emergencies.

At times of natural and human-made disasters, the US Army National Guard and the Air National Guard may be called up by the governor of a state or territory or, in times of war and other national emergencies, by the president. In addition, each of the US Armed Forces (Army, Navy, Marines, Air Force, Coast Guard) have a reserve corps under the control of the federal government that can be deployed to relieve combat troops for select overseas or domestic missions. Most part-time armed forces physician assistant (PA) personnel, collectively referred to as reserve components, hold a full-time civilian job while serving part-time as commissioned officers. In this capacity, they bring a broad array of skills and experiences to their assigned unit, especially in medical service delivery. In turn, the organization and delivery of civilian healthcare are enhanced by the PAs' experience with battlefield trauma, medical and surgical care of diverse populations such as refugees, and medical assets management during natural disasters.

The reserve component makes up about 68% of the strategic medical resources for the Armed Forces, and since the late 1990s, PAs have had an increasing role in medical care and management as commissioned officers.^1^ This is partly due to the military replacing general medical officers who were not board-certified when the requirement was added for physicians to be board-certified.^2^ However, board-certified or not, physicians have historically been difficult to recruit and retain in any component of the military.^3^ With a low inventory of physician leaders, PAs have increasingly served as operational and strategic medical leaders across all military components.^1^,^4^

In 2020, a study of PAs employed in federal roles, including the uniformed services, revealed that about 5,000 PAs work for the government, spanning 20 agencies such as departments, bureaus, administrations, or services. Of PAs employed full-time by the federal government, half had 15 or more years of government service.^4^ The federal employment assessment of PAs did not include those in the reserve component, for the most part considered part-time.

Because US military medical staffing is evolving, the focus of this study was to examine this unique component of uniformed medical service delivery. Interest centered on PAs' versatility spanning civilian and military activities, and the multitude of roles they perform in US society. Specifically, we asked: *What is the profile of PAs who serve in the US Armed Forces reserve components in 2020?*

## MATERIALS AND METHODS

Two data sources were used for information on PAs in the reserve components of the armed services. The first was an informant survey consisting of questions about reserve PAs that was directed to select members of the uniformed services. In 2020, we contacted an active-duty senior PA officer in each of the military branches of the Army, Navy, Air Force, and Coast Guard. The items queried were:

- As of 2020, how many PAs are in the [identified reserve service]?
- What are the characteristics of PAs in the reserve components?

*Potential positions* represented the total number of PAs authorized for that service for 2020. This set of questions was sent to select senior officers and was the second part of a study of PAs employed in federal roles.^4^ The methodology followed the recommended format of key informant surveys developed by Maetas and colleagues.^5^

The second source of data on PAs in the reserve component of the Armed Forces was from the workforce data in the PA Professional Profile compiled by the National Commission on Certification of Physician Assistants (NCCPA).^6^ This survey instrument was developed by drawing on the federal government's Health Resource and Services Administration's (HRSA's) Center for Workforce Studies' minimum data set (MDS) framework. The MDS provides guidelines for collecting fundamental health workforce questions related to demographics, education, and practice characteristics (https://bhw.hrsa.gov/health-workforce-analysis/data). The PA Professional Profile consists of three modules that assess PA demographics, education, and practice characteristics. This survey instrument is presented to PAs through a secure portal on the NCCPA's website.^6^

NCCPA data reflected in this study included responses from PAs who were certified as of December 31, 2019, and reviewed or made updates to their PA Professional Profile between January 1, 2017, and December 31, 2019. By the end of 2019, there were 139,688 certified PAs, and 124,458 provided information for at least a portion of the PA Professional Profile, resulting in an 89.1% overall response rate.

Responses from three questions in the NCCPA platform with specific skip logic were analyzed for the purposes of the study. PAs were asked, “Are you now or have you ever served in the US Armed Forces or US Uniformed Services?” Binomial response options were *yes* or *no*. PAs who indicated *yes* were asked to select all that apply among the choices of Army, Navy, Air Force, Marine Corps, Coast Guard, Public Health Service, and National Oceanic and Atmospheric Administration. From that response, respondents were directed to the following question: “Please select the option below that applies to your current Armed Forces or Uniformed Service status.” The response options were Active Duty, Reserve, National Guard, Retired, and Veteran. Linking these variables with demographic and practice characteristics provided aggregated information about select PA roles in US society. Institutional approval was obtained from the Sterling Institutional Review Board, an independent review board for ongoing evaluation of research studies, for NCCPA PA Professional Profile data in January 2019.

## RESULTS

We first present the results of the senior officer survey. As of 2020, an estimated 1,944 PAs were in the reserve component (Army, Air Force, Navy, Coast Guard, Reserves, Army National Guard, or Air National Guard) with the majority (1,597) in the Army. Some were with battalions, brigades, or equivalent units, and others in medical units or nonclinical roles. Navy PAs can be assigned to the Marines, a landing force of the Navy, but are still included as Navy. If PAs are clinically active in civilian roles, they likely are nationally certified and hold a state license in a patient treatment capacity (Table 1). Some PAs serve in administrative roles such as force commanders, task force liaisons, senior advisors, or academics. A few are on extended active duty.

**TABLE 1.** PAs in the US armed forces reserves, National Guard, and Air National Guard, from a survey of senior military officers in 2020

Unit	Estimated number of PAs	Potential PA positions in reserve component	Male:female ratio
Army National Guard	947	950	65:35
Army Reserves	650	650	60:40
Navy Reserves	49	53	54:46
Air Force National Guard	250	250	58:42
Air Force Reserves	44	54	70:30
Coast Guard Reserves	4	12	50:50
Total	1,944	1,969	N/A

Turning to the second source of data, the NCCPA PA Professional Profile, as of the end of 2019, more than 12,000 PAs self-reported active duty, Army National Guard, Air National Guard, Reserves, retired, or veteran status. This accounts for 10% of all certified PAs. The survey does not ask if they were functioning as PAs in that branch of the military nor does it ask if they were PAs at the time they were in uniform. The survey does not collect years of accumulated military service. Depending on the service branch, 2% to 40% were female (Table 2); clinical experience as a PA averaged 13 to 18 years.

**TABLE 2.** Female PA respondents by branch and military status^7^

Branch	Active duty	National Guard/Air National Guard	Reserve	Retired	Veteran
Air Force	37.2	38.5	31.3	9.4	33.4
Army	22.2	29.5	37	14.5	24.8
Coast Guard	27.9	—	40	14.9	20.9
Marines	2.2	—	15.4	9.5	8.2
Navy	28	—	30.4	9.8	19.3

All figures are percentages.

The civilian medical specialties that PAs bring to the assigned reserve component most often are family medicine, emergency medicine, general surgery, orthopedic medicine and surgery, and internal medicine specialties. PAs are provided a full list of medical and surgical specialties for selection in the PA Professional Profile; however, other than those listed, the numbers are too small for inclusion in Table 3.

**TABLE 3.** PA civilian medical specialty by branch of service (present or past, 2020)^7^

All figures are percentages. Navy PAs may be seconded to the Marines, a branch of the Navy.
	Army	Navy	Air Force	Coast Guard
Family medicine	29.7	27.5	29.3	34.2
Emergency medicine	17.4	13.7	15.5	13.1
General surgery, specialty surgery, and orthopedic medicine and surgery	10.8	12.2	9.5	5.9
Internal medicine—includes general internal medicine and internal medicine specialties	3.6	5.2	4.1	3.6
Occupational medicine	3.2	4	3.4	5

## DISCUSSION

As of end of 2019, about 140,000 PAs in the United States were certified.^7^ During this same time, about 1,500 PAs were on active duty in the Armed Forces.^4^ Concurrently, almost 1,950 PAs were in the multiservice reserve components. Combined, this number represents about 2.5% of all PAs reporting that they had a military occupational status between 2017 and 2020. One-fifth of all reserve component PAs are female. The active-duty component of PAs across all services is about one-third female.^4^ These findings differ from the broader US PA census, in which 69.3% of PAs are female.^7^

PAs in US military uniform are multitasked with a wide range of roles. All are qualified in primary care, occupational health and safety, battlefield medicine (including trauma), public health, sanitation, refugee management, immunization schedules, and leadership as commissioned officers.^1^ Some oversee a broad range of medics and corpsmen assigned to their unit; others work on teams in medical treatment facilities alongside physicians, nurses, and other personnel. All military members are trained in biological and chemical warfare response. Senior officers may command a field unit of physicians, nurses, and various healthcare personnel, including corpsmen and medics. Some reservists may serve as full-time active-duty commissioned officers in roles that are not clinical. For all uniformed PAs, medical education is ongoing; and if qualified, some may opt to attend Staff/War College or the Navy Postgraduate School for a graduate degree.^1^

**FIGURE 1.**
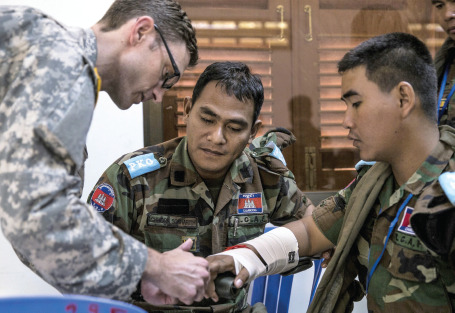
In this 2017 photograph taken in Kampong Speu, Cambodia, Army Capt. Derek Derkacs, PA-C (left), explains to members of the Royal Cambodian Armed Forces how to splint a fracture. Derkacs is a PA for the Boise Veterans Affairs Medical Center and the Idaho Army National Guard's 183rd Aviation Battalion. He traveled to Cambodia to train Cambodian soldiers on lifesaving skills as part of the National Guard's State Partnership Program. Photograph by 2nd Lt. Crystal Farris, Idaho Army National Guard

Unlike career or active-duty officers who relocate periodically, reserve units remain in one location, and familiarity within the group grows. The usual routine for a reservist is to drill 1 weekend a month and 2 weeks a year.^3^ Most perform duty within a unit, although in times of personnel shortages, they may be seconded with individual assignments. For example, in the Coast Guard Reserve, PAs may provide a *locum tenens* role or be assigned to an active-duty function for a few years.^8^ Furthermore, the Army Reserve National Guard and Army Reserve PAs also provide active-duty operational support. This set of orders is separate from deployment orders and provides extended active duty for Army personnel. In addition, Army reserve medical units are periodically activated and deployed in theater, such as in the Middle East or Europe (Figures 1 through 4). All prepare for casualty care, crisis management, and evacuation procedures domestically.^1^

**FIGURE 2.**
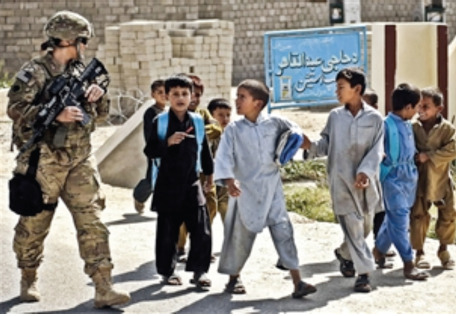
US Air Force Capt. Deana Porter, PA (left), walks with a group of schoolchildren while on a patrol in Mehtar Lam district in Afghanistan's Laghman province in 2011. Porter was assigned to the Laghman Provincial Reconstruction Team, which visited the area to discuss building a second orphanage for children who lost their families.

**FIGURE 3.**
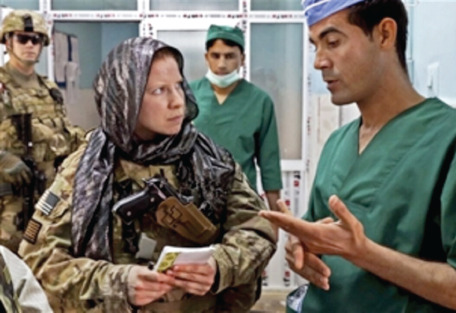
U.S. Navy Lt. jg Laura Cook, PA (left), talks with hospital anesthesiologist Ahmad Jawid Ghafoori during a 2013 meeting at Farah City Hospital in Afghanistan's Farah province. Cook is assigned to Provincial Reconstruction Team Farah.

**FIGURE 4.**
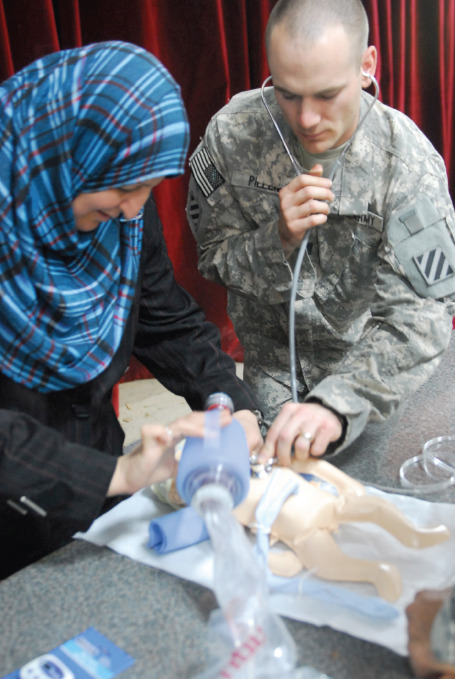
Capt. John Pillen, a PA with the 1st Battalion, 76th Field Artillery Regiment, 4th Advise and Assist Brigade, 3rd Infantry Division, US Division, teaches resuscitation techniques to an Iraqi neonatal nurse during a class at Anbar Provincial Government Center. Photo by Staff Sgt. Tanya Thomas

Most Army National Guard, Air National Guard, and Reserve PAs are assigned to battalions or equivalent contingent units; others serve in medical units. All are nationally certified and hold a state license in a patient treatment capacity. Depending on the service branch, 8% to 35% are female, and clinical experience averages 13 to 18 years. The civilian specialties that PAs typically bring to the assigned units are family medicine, emergency medicine, surgery, orthopedics, and internal medicine. A growing PA presence across all the services, both active duty and reserve, has been underway since 1990.^9^ Their utility and career is ever-changing due to their growing presence in US medicine, as well as the changing landscape of modern healthcare.

Military medicine and staffing are of interest to health workforce planners both in the uniformed services as well as in the civilian sector.^10^ The use of PAs on active duty and in reserve component roles includes a number of workforce strategies intended to expand their responsibility and bypasses state regulations that may constrain their independence to provide optimal medical services.^11^ In addition, the Armed Forces of the United States and their North Atlantic Treaty Organization (NATO) partners continue to be engaged around the world in regions of conflict. Consequently, combat casualty care is a central focus of military medical units and may include personnel from other nations.^12^ Accordingly, present deployment requirements demand the sustaining of a significant number of ready and capable trauma surgical providers to optimize outcomes for injured combat casualties.^13^ A call-up of the Army National Guard, Air National Guard, and Army Reserve medical personnel, including PAs, occurred across some states during the 2020-21 COVID-19 pandemic.^14^,^15^ The influence and experience of the US military during the crisis were noted.^16^ This involved adopting new and novel medical advances in pulmonary medicine, efficiently implementing medical solutions to overcrowded critical care units, detecting, and treating acute respiratory syndromes, and management of the surge of patients by setting up field hospitals along with the Federal Emergency Management Agency in select regions of the country.^16^ In field hospital-like settings, the use of Army and Air Force PAs as critical care medical officers was notable (Table 4).^17^

**TABLE 4.** The US Army Reserve medical team response during the pandemic

The SARS Cov-2 pandemic has taxed medical systems worldwide. Acute care hospitals and critical care beds quickly filled. Because healthcare demand exceeded available resources at numerous healthcare systems in the United States, county and cities requested assistance from their state's office of emergency management. When state assets were unable to support local systems, states requested assistance through the Federal Emergency Management Agency (FEMA). Through the Defense Support to Civil Authorities (DSCA), members of active duty, reserve, and national guard troops in the Army, Navy, and Air Force were requested to fill staffing of the FEMA pandemic disaster response system.
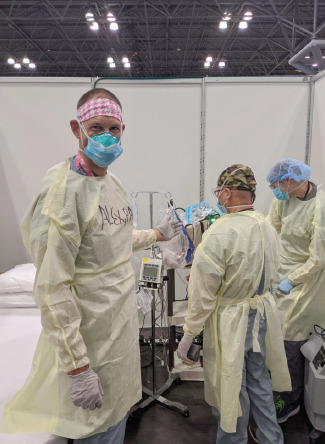
Capt. Alex Merkle, DSc, PA-C, received an unexpected phone call in April 2020 while home in California. The caller introduced himself as the new unit commander in the US Army Reserve and directed him to book a flight the following day so he could join a newly formed Urban Augmentation Medical Task Force (UAMTF) staffed by an Army Reserve medical unit. Within 36 hours, Merkle had a copy of military mobilization orders (which usually take weeks to months to generate) and was aboard a nearly empty plane heading for the East Coast.
The UAMTF group met outside Washington, D.C., and began assembling a team of 85 Army Reservists, based on the FEMA disaster medical assistance team template used for natural disaster response. FEMA teams often include physicians in family medicine, dentists, and mental health professionals who are called upon after natural disasters. The team moved to an intermediate staging area an hour outside New York City, and the following day was taken by charter bus into an empty, postapocalyptic appearing Manhattan.
The UAMTF team was quickly incorporated into the recently opened federal medical center in the Javits Convention Center. Each of the 85 members were assigned individual hotel rooms to minimize disease transmission. As a fellowship-trained critical care PA, Merkle was assigned to the ICU in the convention center (see photo). The majority of the 500 beds were for patients who no longer needed hospital-level care, but still required several more days of convalescence and supplementary oxygen before being discharged. Most of the patients did well and went home within a few days, but a small number would clinically deteriorate and were taken to the ICU center for closer monitoring. Many of those in the ICU went on to require intubation due to poor oxygenation. Because of advanced training as a medical unit, the critical care medical staff were able to adapt and change the medical mission as frequently as every 12 hours as patient surges and equipment changed. Merkle reported that the hardest part of his time in the Javits ICU was setting up video calls with rapidly deteriorating patients so they could see their family before intubation, and often for the last time.
This example of a citizen-soldier healthcare provider represents one of the many medical personnel who took part in one of the largest and fastest mobilizations of healthcare resources in US history. Such adroitness and quick assembly of a reserve medical team is due in large part to military training in which senior leadership creates strategy and those who implement the mission determine the correct tactics for the operational environment.

Disclaimer: The views and opinions are those of the authors and do not necessarily reflect those of the Department of Defense or US Army. Information regarding the military mobilization and COVID may be inaccurate, given the rapidly evolving mission set and quick decision-making required for the national disaster pandemic response.

Although this human resource study offers a basic inventory of PA assets in the reserve components, spread over five military services, it identifies only a small part of what is known about their roles, responsibilities, and employment nature. For the healthcare workforce planner, medical organization analyst, and labor economist, a more granular picture has yet to be understood in terms of role delineation, duration of military service, rank and pay structure, skills, job description, career arc, as well as whether the PA is in an active or inactive reserve status.^11^,^18^ How part-time uniformed PAs fit in with a larger medical team of physicians, nurses, corpsmen and medics, and administrators is beyond the scope of this study but of interest to military medical planners. The key to disaster readiness is a strong military-civilian partnership.^19^ This study of military PAs was undertaken with the intent to set the stage for a more refined examination of the dual employment role of PAs.^20^

## LIMITATIONS

As with any survey study, the data collected through the NCCPA PA Practice Profile may be affected by recall bias and memory limitations. Additionally, the PA Practice Profile does not collect information on individual ready reserve (inactive) status. The questions posed to the various sources of information on PAs in the reserve components were brief and details of answers were set aside for more granular examination of roles and duality of PA careers.^20^

## CONCLUSION

The US military draws on PAs to provide healthcare to diverse populations, and the reserve components of the Armed Forces mirror this active-duty mission. PAs have become a critical element of reserve components as medical officers by providing a flexible and adaptable resource for military missions, both in domestic and international settings. The difficulty of recruiting board-certified physicians and surgeons has compelled the uniformed services to adapt and use PAs in unprecedented ways. What these PAs do in their roles and their effectiveness is where the next focus of research is recommended.
